# Development of a Sensitive SERS Method for Label-Free Detection of Hexavalent Chromium in Tea Using Carbimazole Redox Reaction

**DOI:** 10.3390/foods12142673

**Published:** 2023-07-11

**Authors:** Limei Yin, Heera Jayan, Jianrong Cai, Hesham R. El-Seedi, Zhiming Guo, Xiaobo Zou

**Affiliations:** 1 Key Laboratory of Modern Agricultural Equipment and Technology of Ministry of Education, School of Agricultural Engineering, Jiangsu University, Zhenjiang 212013, China; yinlm6@163.com (L.Y.); jrcai@ujs.edu.cn (J.C.); 2 China Light Industry Key Laboratory of Food Intelligent Detection & Processing, School of Food and Biological Engineering, Jiangsu University, Zhenjiang 212013, China; heerajayan93@outlook.com (H.J.); zou_xiaobo@ujs.edu.cn (X.Z.); 3 Pharmacognosy Group, Department of Pharmaceutical Biosciences, BMC, Uppsala University, Box 591, SE 751 24 Uppsala, Sweden; hesham.el-seedi@fkog.uu.se; 4 International Joint Research Laboratory of Intelligent Agriculture and Agri-Products Processing, Jiangsu University, Zhenjiang 212013, China

**Keywords:** SERS detection, chromium contamination, tea sample, carbimazole hydrolysate, Au@Ag nanoparticles

## Abstract

Tea plants absorb chromium-contaminated soil and water and accumulate in tea leaves. Hexavalent chromium (Cr^6+^) is a very toxic heavy metal; excessive intake of tea containing Cr^6+^ can cause serious harm to human health. A reliable and sensitive surface-enhanced Raman spectroscopy (SERS) method was developed using Au@Ag nanoparticles as an enhanced substrate for the determination of Cr^6+^ in tea. The Au@AgNPs coated with carbimazole showed a highly selective reaction to Cr^6+^ in tea samples through a redox reaction between Cr^6+^ and carbimazole. The Cr^6+^ in the contaminated tea sample reacted with methimazole—the hydrolysate of carbimazole—to form disulfide, which led to the decrease in the Raman intensity of the peak at 595 cm^−1^. The logarithm of the concentration of Cr^6+^ has a linear relationship with the Raman intensity at the characteristic peak and showed a limit of detection of 0.945 mg/kg for the tea sample. The carbimazole functionalized Au@AgNPs showed high selectivity in analyzing Cr^6+^ in tea samples, even in the presence of other metal ions. The SERS detection technique established in this study also showed comparable results with the standard ICP-MS method, indicating the applicability of the established technique in practical applications.

## 1. Introduction

Chromium has been widely employed in various industries and has become a major threat to the environment and human health [1,2]. The heavy metal chromium enters the metabolic and digestive system of the human body through contaminated foods and causes various health effects based on the valence state. Chromium exists stably in the environment as two different oxidation states, the trivalent chromium (Cr^3+^) cation and the hexavalent chromium (Cr^6+^) anion; however, their chemical properties are markedly different, and Cr^6+^ is far more dangerous than Cr^3+^ [3]. The presence of an appropriate amount of Cr^3+^ in the human body is non-toxic and beneficial to human metabolism and health. Cr^3+^ is a necessary trace element for glucose, fat, and protein metabolism in mammals [4]. However, the presence of Cr^6+^ in food increases the risk of cancer and mutation in the human body [4]. Cr^6+^ mainly exists in the industrial discharge of dyes and tanneries. If the tea plants were grown in chromium-contaminated soil or irrigated with chromium-contaminated water, the tea plants continued to absorb and enrich chromium pollutants, eventually leading to the accumulation of chromium pollutants in the tea. The safety assessment of agricultural products as a key important food source of heavy metals is of great significance to ensuring food safety.

Tea (*Camellia sinensis*) is the most popular flavored and functional beverage worldwide. Tea is particularly rich in polyphenols, amino acids, caffeine, and other effective components, with lowering blood lipids and blood sugars, anti-inflammatory, antibacterial, antioxidant and other health effects, which are thought to contribute to the health benefits [5]. In the international trade of tea, the primary problem is safety, in which respect the residue of risk substances, especially heavy metal pollutants, has aroused great concern. The maximum residual limit for chromium in tea is 5 mg/kg according to a standard issued by the Ministry of Agriculture of China [4]. Thus, a rapid, efficient, and accurate quantitative detection method is essential to prevent chromium-contaminated from entering the tea planting and production process from different sources. Nowadays, the determination methods of Cr^6+^ mainly include atomic absorption spectroscopy [6], liquid chromatography [7], immunoassay [8], fluorescence spectroscopy [9], and inductively coupled plasma mass spectrometry (ICP-MS) [10]. However, these techniques usually require sophisticated equipment, skilled personnel, and long and tedious sample preparation [11]. Therefore, a simple, sensitive, and reliable method for the determination of Cr^6+^ would be very useful to ensure the safety and quality of food products [12].

Surface-enhanced Raman spectroscopy (SERS) is a reliable, sensitive, and non-destructive method popular for the detection of trace amounts of contaminants from complex matrices. In the past decade, SERS detection has been extensively applied in several fields including environmental monitoring, food safety, and pharmaceutical analysis [13]. The increasing use of SERS substrates has prompted researchers to use green reducing agents to prepare nanoparticles [14,15]. Carbimazole (Ethyl 3-methyl-2-thioimidazoline-1-carboxylate) is the main functional component of an oral drug for the treatment of thyroid diseases. After entering the human body, it can be hydrolyzed into methimazole in the acidic environment of the stomach [16]. By simulating the human stomach environment, acidic conditions can be generated in an aqueous solution. Tannins, the green natural, tannins have a strong reducing ability and can be employed for the synthesis of gold-silver core–shell nanoparticles (Au@AgNPs) at room temperature. In the reaction solution, carboprazole was adsorbed on the surface of Au@AgNPs, and its Raman signal was greatly enhanced due to the “hot spot” between the nanoparticles [17]. 

**Figure 1:**
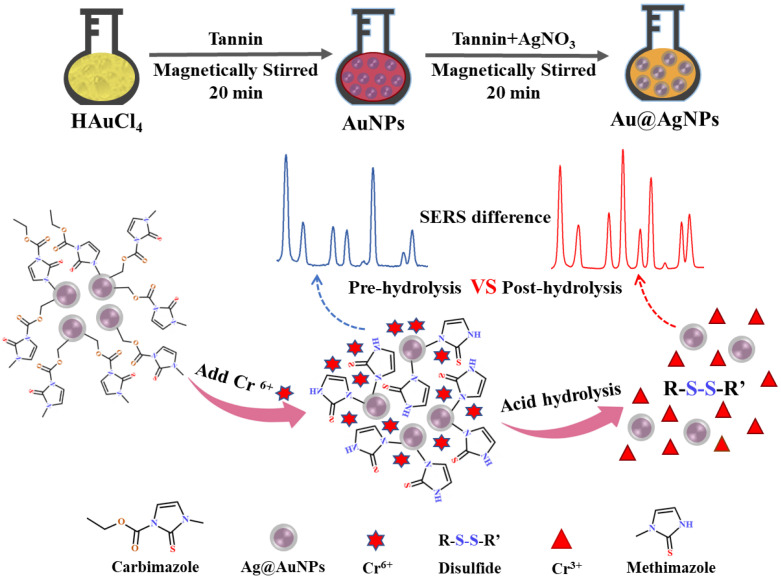
Schematic illustration of the mechanism of SERS detection of hexavalent chromium.

In this study, a convenient and sensitive SERS method for the detection of Cr^6+^ in tea was developed by carbimazole redox reaction. The main objectives of this study are: (1) to synthesize a SERS substrate of bimetallic core–shell nanoparticles (Au@AgNPs) to acquire a higher and stable Raman signal; (2) to obtain the highest Raman enhancement factor by optimizing the tannin with different volumes and concentrations of HAuCl_4_ and AgNO_3_; (3) to elucidate the mechanism of the decrease in Raman characteristic peak intensity caused by the redox reaction between Cr^6+^ and methimazole, the hydrolysate of carbimazole; and (4) to establish the linear quantitative equation of Raman intensity and the concentration of Cr^6+^ and to analyze the specific selectivity of carbimazole to Cr^6+^. The schematic diagram of SERS detection of Cr^6+^ is illustrated in Figure 1. 

## 2. Materials and Methods

### 2.1. Materials

Tannin (92%), 4-mercaptobenzoic acid (4-MBA, 97%), chloroauric acid tetrahydrate (HAuCl_4_·4H_2_O, 99%), silver nitrate (AgNO_3_, 99.8%), carbimazole (C_7_H_10_N_2_O_2_S, 98%), sodium chloride (NaCl, 99%), potassium carbonate (K_2_CO_3_, 99%), and nitric acid (HNO_3_, 68%) were obtained from National Pharmaceutical Group Chemical Reagents Company (Beijing, China). Standard solution of mercury (Hg), manganese (Mn), nickel (Ni), lead (Pb), copper (Cu), cadmium (Cd), iron (Fe), arsenic (As), sodium (Na), and chromium (Cr), were procured from Siyuan Chemical Glass Co., Ltd. (Zhenjiang, China), and all their purities were greater than 97%. Ultrapure water was used for all the experiments. All reagents used in the study were analytical grade unless stated otherwise.

### 2.2. Instruments 

Au@AgNPs were characterized using Ultraviolet–Visible (UV–Vis) absorption spectroscopy (Agilent Technologies Inc., Palo Alto, CA, USA), Tecnai 12 transmission electron microscope (TEM) (Philips, Amsterdam, Holland,) and Fourier–transform infrared (FTIR) spectroscopy (Beijing Rayleigh Analytical Instrument Co., Ltd., Beijing, China). Microwave digestion instrument (MARS 6) (CEM, Charlotte, NC, USA) was used for the digestion of food samples. The confocal micro-Raman imaging spectrometer (XploRA PLUS, HORIBA, MPL, France) was employed to collect Raman spectra from the samples. The employed excitation wavelength and objective lens were 785 nm and 50× (Spot size: 1.28 μm), respectively. The ICP-MS (Thermo Fisher Scientific, X Series 2, Waltham, MA, USA) analysis was performed to detect Cr^6+^ in the spiked tea digestions.

### 2.3. Synthesis of SERS Substrate Au@AgNPs

Synthesis of AuNPs: Gold nanoparticles were prepared by utilizing tannins as a reducing agent [18]. Briefly, 1 mL of 10 mM tannin was added to a 50 mL beaker containing 18.5 mL of ultrapure water under stirring conditions (500 rpm), followed by the addition of 500 μL of 1% HAuCl_4_·4H_2_O. The total volume of the reaction mixture was about 20 mL, and the reaction was allowed to continue for 20 min. The pH of the solution was measured at 6 due to the acidic nature of the tannin. Another batch of nanoparticles was also prepared by adding HAuCl_4_·4H_2_O (1%) after adjusting the pH of the solution to 7 with K_2_CO_3_ solution (0.2 M). 

Synthesis of Au@AgNPs: The prepared AuNPs were coated adding a silver layer by reducing AgNO_3_ using tannins as a reducing agent [19]. In a 25 mL round-bottom flask, 9.5 mL of prepared AuNPs colloidal solution was stirred at 500 rpm. Then, 250 μL of 10 mM tannin solution and 250 μL of 10 mM AgNO_3_ solution were sequentially added to the solution with constant stirring for 30 min at room temperature. The formation of the core–shell structure was indicated by the change in colour of the solution from wine red to orange. 

Synthesis optimization: It was found that the volume of HAuCl_4_ and AgNO_3_ during the preparation of Au@AgNPs greatly affects the size and concentration of nanoparticles formed in the solution, which will, in turn, affect the enhancement capabilities of the prepared substrate. In order to obtain high enhancement abilities, the volume of the two reactants (HAuCl_4_ and AgNO_3_) was optimized. The synthesis of AuNPs involved varying the volume of HAuCl_4_ from 100 to 600 μL with an increment of 100 μL. The enhancement of the AuNPs was evaluated using 4-MBA (10^−3^ M) as a Raman signal probe. Meanwhile, the synthesis of Au@AgNPs involved varying the volume of AgNO_3_ from 100 to 600 μL with an increment of 100 μL. The UV–Visible spectra of the synthesized Au@AgNPs substrates were collected to better understand the difference in surface plasmon resonance. Further, the enhancement of the Au@AgNPs was evaluated using 4-MBA (10^−5^ M) as a Raman signal probe.

### 2.4. Tea Sample Preparation

The black tea sample was purchased from the supermarket in Zhenjiang, Jiangsu. After grinding it into powder, the tea powder (0.2 g) was weighed and added to the microwave digestion tank. Concentrated HNO_3_ (65%, 8 mL) and the Cr^6+^ standard solution was added to the tea sample before digestion (1 h). The digestion tanks were put in an acid extractor (130 °C, 60 min), cooled, and then digested in a microwave digester following the standard operating procedure with some modifications. Finally, a light green clarified solution was obtained without any solid residue [20]. The digested samples were degassed by sonication (100 °C, 10 min), and the inner cap was rinsed with a little water. Then the resulting digestion solution was filtered into 50 mL volumetric flasks with syringe filter (diameter 33 mm, pore size 0.22 μm) and filled up to the mark with ultrapure water to obtain the final solution to be assayed. A total of 8 samples spiked with various amounts of Cr^6+^, including 100, 80, 60, 40, 20, 10, 5 μg L^−1^, and blank, were used for the analysis. The prepared samples were also used for ICP-MS and SERS analysis.

### 2.5. Detection of Cr^6+^ in Tea Samples

The concentration of Cr^6+^ in tea samples was analyzed using Au@AgNPs in the presence of carbimazole solution. First, 40 μL of Au@AgNPs solution and 5 μL carbimazole solution (10 mM in chloroform) were mixed together for 10 min. Then, digested tea samples were added and mixed thoroughly. Finally, 5 μL NaCl (10 mM) was introduced to the solution to aggregate the nanoparticles in the solution which increases the local hotspot and improves the Raman signal intensity [21]. A piece of tin foil tape measuring approximately 5 cm in length (with a thickness of 0.09 mm and width of 20 mm) was carefully affixed flat onto a glass slide. The reaction solution with a total volume of 50 μL was allowed to sit undisturbed for 10 min. Then, 1 μL of the solution was gently placed onto the surface of the tin foil tape, shaping the droplet into a round shape as much as possible. It was left to air dry, and the SERS spectra were subsequently collected from within the dried droplet [22]. All spectra were collected using a confocal micro-Raman imaging spectrometer equipped with a 785 nm excitation laser (100% power). The total acquisition time was set at 5 s. Five spectra were randomly collected from the droplets of each sample, and the average values of the spectra of each concentration were taken as the final spectral data. The characteristic peaks for the Cr^6+^ were identified by comparing the obtained spectra with that of the Raman spectra of carbimazole powder. The standard curve for the quantitative analysis was obtained using digested tea samples containing different concentrations of Cr^6+^ and digested tea solution without Cr^6+^ was used as blank. The spectral intensity and intensity ratio of specific peak positions were taken into consideration to establish the calibration curve. Subsequently, the quantitative ability and accuracy were analyzed based on the calibration curve.

### 2.6. Specific Selectivity and Spike Recovery for Cr^6+^ Detection in Tea

According to the previous literature [23,24], common metal ion pollutants in tea include Hg^2+^, Mn^2+^, Ni^2+^, Pb^2+^, Cu^2+^, Cd^2+^, Fe^3+^, As^3+^, and Na^+^. To validate the selectivity of carbimazole in detecting Cr^6+^, the change of Raman intensity at the characteristic peak (595 cm^−1^) was compared when these metal ions were added to the system at an equal concentration (100 μg/L). Additionally, the experiment was repeated 3 times for the spiked tea sample (5 μg/L), and the spectra of 15 different points were obtained on each prepared detection solution to analyze the repeatability of the method. The relative standard deviation (RSD) of the spectral intensity at the characteristic peak was calculated to analyze the repeatability of the detection method. the reproducibility of the method

The quantitative data obtained from the SERS analysis were compared against the standard ICP-MS method [25]. Tea samples with different concentrations of Cr^6+^ (2.5, 5, 10, 20 mg/kg) were used for ICP-MS analysis with triplicates, and the recovery rate and RSD values were calculated for each sample. Thus, the practical applicability of the developed method for the detection of Cr^6+^ was confirmed.

## 3. Results

### 3.1. Synthesis of Au@AgNPs and Optimization

**Figure 2:**
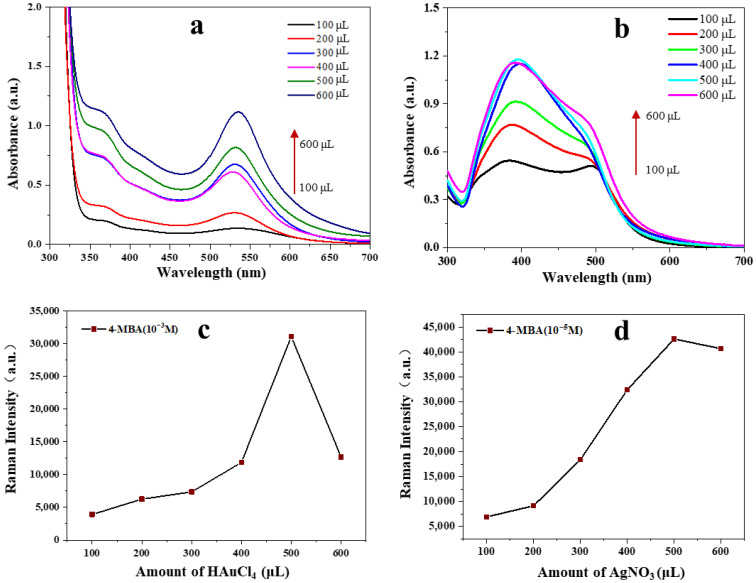
(**a**) UV spectra of AuNPs synthesized by tannin with different volumes of HAuCl_4_; (**b**) UV spectra of Au@AgNPs reduced by tannin with different volumes of AgNO_3_; (**c**) SERS enhancement of AuNPs synthesized using different volumes of HAuCl_4_; (**d**) SERS enhancement of Au@AgNPs synthesized using different volumes of AgNO_3_.

Tannins are naturally occurring polyphenols that can act as an ideal reductant in the synthesis of nanoparticles. Tannins were responsible for the reduction of HAuCl_4_, resulting in the formation of stable Au NPs which then serve as a seed to induce Ag NPs synthesis. The change in colour of the colloidal nanoparticle from wine red to orange indicated the formation of the core–shell structure. The surface plasmon resonance of the prepared nanoparticles with different amounts of precursors exhibited a change in surface plasmon resonance, as shown in Figure 2a. As the volume of HAuCl_4_ increased, the colour of the nanoparticle solution gradually changed, and when the volume of HAuCl_4_ reached 600 μL, the synthesized nanoparticle solution appeared turbid and exhibited aggregation and precipitation after 3 days of storage [26]. The UV–Vis spectra of AuNPs were shown in Figure 2a, and the peak at 520 cm^−1^ increased gradually and a slight red shift occurred as the volume of HAuCl_4_ increased. This observation suggested that the particle size of Au NPs increased [27]. The enhancement effect of AuNPs on the Raman reporter molecule 4-MBA (10^−3^ M) was also used to optimize the amount of HAuCl_4_. As shown in Figure 2c, the Raman intensity at 1074 cm^−1^ was the greatest when the amount of HAuCl_4_ was 500 μL. It was attributed to AuNPs with larger particle size can produce strong localized surface plasmon resonance. However, as the amount of HAuCl_4_ continued to increase (600 μL), the Raman intensity decreased. This is because the larger particle size leads to the instability of the Au NPs. Considering the stability of storage and Raman enhancement effect, the optimal volume of HAuCl_4_ was selected as 500 μL.

To increase the Raman intensity, the growth of the Ag shell on the Au NPs was carried out. The amount of AgNO_3_ was optimized because it affected the thickness of the Ag shell and thus affected the Raman intensity. As shown in Figure 2b, as the amount of AgNO_3_ increased, the Ag absorption peak around 400 nm gradually intensified, while the Au absorption peak near 520 nm weakened rapidly or even vanished entirely. The Raman intensity of 4-MBA (10^−6^ M) at 1584 cm^−1^ was also used to determine the optimal amount of AgNO_3_. As shown in Figure 2d, the Raman intensity reached its maximum when the volume of AgNO_3_ was 500 μL. However, the Raman intensity weakened slightly when the amount of AgNO_3_ increased to 600 μL, which was due to the 4-MBA signal transmission being hindered by a thicker Ag shell. Therefore, the optimal amount of AgNO_3_ was 500 μL.

### 3.2. Characterization of Au@AgNPs

**Figure 3:**
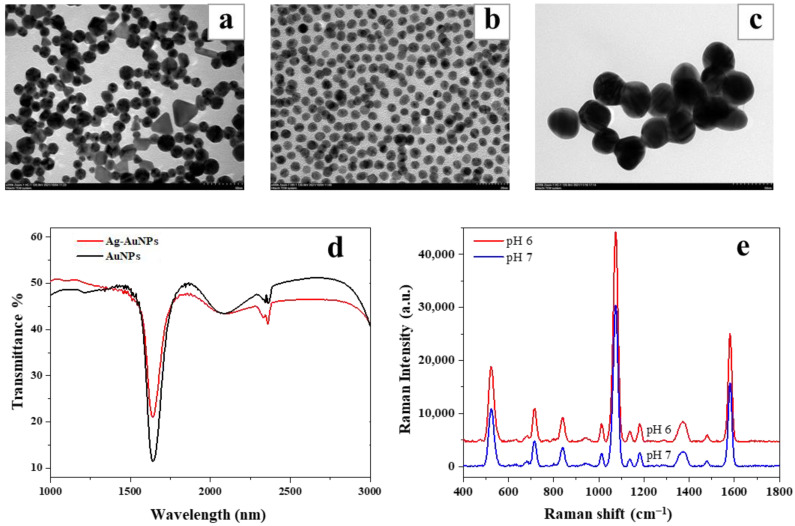
TEM image of AuNPs synthesized under pH 7 (**a**) and pH 6 (**b**); (**c**) TEM image of Au@AgNPs synthesized under pH 6; (**d**) FT-IR spectra of AuNPs and Au@AgNPs; (**e**) SERS enhancement of Au@AgNPs synthesized under pH 6 and pH 7 conditions.

The morphology and distribution of nanoparticles were observed and measured by TEM. The size and morphology of the prepared nanoparticles changed based on the pH of the solution as shown in Figure 3a,b. The nanoparticles synthesized under pH 7 were aggregated and non-uniform compared to AuNPs synthesized under pH 6. The colloidal solution contained triangular nanoparticles, polygonal nanoparticles, nanorods, and irregular particles; however, the round nanoparticles account for the majority. Figure 3c shows the TEM image of Au@AgNPs synthesized under pH 6, which indicated that the formed particle had a particle size of around 10–30 nm. Since the shape and size of the nanoparticles formed under pH 6 conditions showed more uniformity, pH 6 was selected for further synthesis and applications. 

For the FTIR characterization, spectra were collected from a 10 μL of drop cast nanoparticle solution, and the spectra displayed a characteristic peak at 1400 cm^−1^ for AuNPs (Figure 3d). Further, the silver coating caused a decrease in the peak intensity at 1400 cm^−1^ and a slight shift. The enhancement effects of Au@AgNPs synthesized under pH 6 and pH 7 conditions were also compared, and the obtained spectra are shown in Figure 3e. The calculated enhancement factors for Au@Ag nanoparticles synthesized under pH 6 and pH 7 conditions were 3.56 × 10^5^ and 2.39 × 10^5^, respectively. The Au@Ag further prepared by AuNPs synthesized at pH 6 had a better enhancement effect, which was closely related to the better uniform dispersion of AuNPs under this condition.

### 3.3. Quantitative Detection of Cr^6+^ in Tea Samples

**Figure 4:**
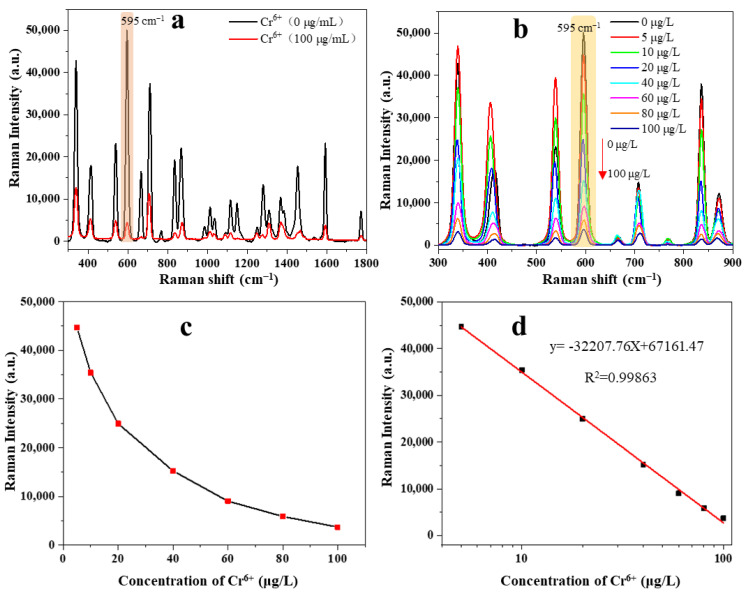
(**a**) SERS spectra of Cr^6+^ (0 µg/L) and Cr^6+^ (100 µg/L); (**b**) SERS spectra of Cr^6+^ in tea at different concentrations ranging from 0 to 100 µg/L; (**c**) the SERS intensity at 595 cm^−1^ at various concentration of Cr^6+^; (**d**) calibration curve between the SERS intensity at 595 cm^−1^ and logarithm of the concentration of Cr^6+^.

The detection and quantitative analysis of Cr^6+^ in tea samples were obtained based on the redox reaction between carbimazole and Cr^6+^. The hydrolytic product (methimazole) of carbimazole was adsorbed on the surface of Au@Ag via Ag-S and Ag-N bonds, and its Raman signal was greatly enhanced. At the same time, the aggregation of these functionalized substrates caused by NaCl also resulted in significant SERS enhancement [16]. In order to identify the characteristic peaks of Cr^6+^, the spectra of high concentration of Cr^6+^ solution (100 μg/L) were compared with the blank tea sample (Cr^6+^ = 0 μg/L) (Figure 4a). Carbimazole was hydrolyzed to methimazole under acidic media that causes carbimazole to have a similar Raman spectrum to methimazole. The strongest characteristic peak was shown at 595 cm^−1^ due to the enhancement of Au@AgNPs on methimazole. The presence of Cr^6+^ causes a redox reaction between Cr^6+^ and methimazole, forming a disulfide compound and resulting in a decrease in Raman intensity at 595 cm^−1^. Therefore, the quantitative analysis of Cr^6+^ can be realized by using the change of Raman intensity at 595 cm^−1^. The main peaks of the methimazole contribution are listed in Table 1. As can be seen from Figure 4a, the characteristic peak at 595 cm^−1^ was attributed to the vibration of the C–N–S bend.

The SERS spectra of different concentrations of Cr^6+^ ranging from 0 to 100 µg/L were obtained and are shown in Figure 4b. The Raman intensity at 595 cm^−1^ significantly decreased with increasing Cr^6+^ concentration in the tea samples (Figure 4c) as the methimazole was reduced to disulfide. The calibration curve of Cr^6+^ was obtained using the relationship between the Raman intensity at 595 cm^−1^ and the logarithm of the concentration of Cr^6+^ (Figure 4d). The calibration curve showed a good linear relationship in the range of 5~100 μg/L (R^2^ = 0.99863). The linear quantitative relationship was described by the equation y = −32,207.76X + 67,161.47. Furthermore, the limit of detection (LOD) was defined as the concentration of Cr^6+^, resulting in a 3% decrease in Raman intensity related to the blank tea sample (0 μg/L) [28]. According to the established equation, the LOD was calculated to be 3.78 μg/L, indicating that the immunosensor had a good sensitivity. Due to the dilution factor of 250 times in the pretreatment of tea samples, the detection range of Cr^6+^ in tea sample was 1.25~25 mg/kg, with a LOD of 0.945 mg/kg, which was much lower than the recommended tolerable level (5 mg/kg) of Cr^6+^ in tea.

### 3.4. Specific Selectivity and Recovery for Cr^6+^ Detection in Spiked Tea

**Figure 5:**
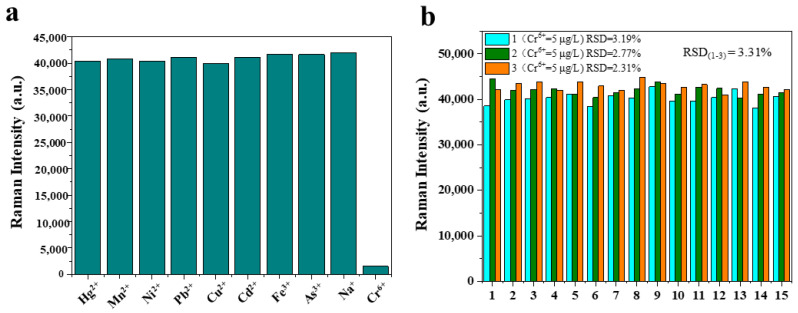
(**a**) Specificity of SERS detection for Cr^6+^ in tea when the concentration of all the ions was 100 µg/L; (**b**) relative standard deviation (RSD) of three repeated experiments and 15 random points in the detection solution at 595 cm^−1^.

For evaluating the specificity of the SERS method for Cr^6+^ analysis, some metal ions commonly found in tea, including Hg^2+^, Mn^2+^, Ni^2+^, Pb^2+^, Cu^2+^, Cd^2+^, Fe^3+^, As^3+^, Na^+^, and Cr^6^, were selected as interferences [10]. The Raman intensity of the peak at 595 cm^−1^ was shown in Figure 5a, and the results showed that only the Cr^6+^ (100 µg/L) have a specific redox reaction with the hydrolysate of the carbimazole, resulting in the decrease in the Raman intensity at the 595 cm^−1^ peak. Therefore, the presence of other ions does not interfere with the quantitative detection of Cr^6+^. 

The experiment was repeated three times at the same concentration (Cr^6+^ = 5 µg/L) to verify the reproducibility. For each detection solution, 15 random points were chosen to obtain the Raman intensity at peak 595 cm^−1^. As shown in Figure 5b, in each experiment, the RSD value of 15 points were all less than 5%, indicating that the detection solution had good uniformity. Similarly, the RSD value of the three repeated experiments was less than 5%, indicating that the detection method had a good reproducibility.

Further the quantitative results obtained from SERS detection were validated against standard ICP-MS, and the results are shown in Table 2. The recovery rates for the SERS method ranged from 91.62% to 104.84% (RSD ≤ 3.07%), while the recovery rates of the ICP-MS method ranged from 97.65% to 103.86% (RSD ≤ 1.53%). Comparative analysis indicates that the SERS method demonstrates higher accuracy and good reproducibility in detecting Cr^6+^ content in tea leaves. 

## 4. Discussion

The present study demonstrated the development of a sensitive and reliable method for the quantitative detection of Cr^6+^ in tea samples. The method relied on the redox reaction between Cr^6+^ and carbimazole, leading to a decrease in the intensity of the Raman peak at 595 cm^−1^. This study provided valuable insights into the potential application of SERS technology as a rapid and label-free detection technique for Cr^6+^ in tea. The results indicated that the Raman intensity of the characteristic peak at 595 cm^−1^ was inversely correlated with the concentration of Cr^6+^. Moreover, the relationship between the Raman intensity at 595 cm^−1^ and the logarithm of the concentration of Cr^6+^ was linear. The LOD (3.78 μg/L) of Cr^6+^ calculated using this approach indicated the sensitivity of the developed method. Compared with the previous study [29] using photoelectrochemical to detect Cr^6+^ (LOD = 0.01 μM) in the environment, the proposed method exhibited similar sensitivity and simpler detection operation.

Another key aspect investigated in this study was the selectivity of carbimazole towards Cr^6+^ in the presence of other metal ions. A specific selection of Cr^6+^ by carbimazole was demonstrated, highlighting the potential applicability of the developed method for real tea samples, which may contain various metal ions as contaminants. To validate the accuracy and reliability of the developed SERS method, a comparison was carried out with the standard method (ICP-MS). The reproducibility, accuracy, and recovery rate of the SERS technique were thoroughly analyzed. The results showed that the SERS method exhibited comparable performance to the ICP-MS method in terms of accuracy and recovery rate, indicating its suitability for practical applications.

The findings of this study exhibited the potential of SERS technology for the sensitive and label-free detection of Cr^6+^ in tea samples. The advantages of SERS, such as its high sensitivity, non-destructivity, and minimal sample preparation requirements, make it an attractive alternative to traditional analytical techniques. The established SERS method offers a convenient and sensitive approach for the determination of Cr^6+^, which is of great importance considering the potential health hazards associated with its presence in tea. The results obtained in this study contribute to the growing interest in the application of SERS technology for the detection of contaminants in food, and it paves the way for further research.

## 5. Conclusions

In this study, a highly sensitive nano-SERS substrate was developed for the detection of Cr^6+^ in tea samples. The Au@AgNPs with uniform particle size distribution and good enhancement effect was synthesized using tannin. The combination of the specific redox reaction of carbimazole and Cr^6+^ and NaCl-induced aggregation of nanoparticles enhanced the EF value of Au@AgNPs to 3.56 × 10^5^. Compared with AuNPs, the EF of Au@AgNPs was two orders of magnitude higher, thus improving the sensitivity of Cr^6+^ detection. Quantitative analysis showed a linear relationship between the Raman intensity of the characteristic peak at 595 cm^−1^ and the logarithm of the Cr^6+^ concentration. The LOD of this proposed SERS method was 3.78 μg/L, and the detection range was established to be 5~100 μg/L. Due to the 250-fold dilution during tea sample processing, the detection range of Cr^6+^ in tea sample was 1.25~25 mg/kg, with a LOD of 0.945 mg/kg. Additionally, the proposed method showed high specificity, even in the presence of other metal ions, and good reproducibility in detecting Cr^6+^ in the tea sample. In particular, it showed good accuracy (recovery rates ranged from 91.62% to 104.84%) and precision (RSD ≤ 3.07%) in the recovery experiment, and the obtained results were validated against ICP-MS. Conclusively, this method has great potential for rapid and label-free detection of Cr^6+^ in tea.

## Figures and Tables

**Table 1:** Raman band assignments for peak obtained during Cr^6+^ detection.

Raman Shift (cm^−1^)	Band Assignment
412.63	Ring rotation, C–N–S bend
539.54	S=S stretching
595.85	C–N–S bend
708.48	Ring rotation, CH(NH) bend
872.67	Ring rotation, CH(NH) bend, C–N–S bend
1014.35	C–N stretching, CH(NH) bend
1370.14	C–N stretching, bend and rotation
1469.72	C–S stretching, CN stretching, NH bend
1593.76	C–C stretching, CH(NH) bend

**Table 2:** Comparison between the SERS method (this study) and ICP-MS quantitative detection of Cr^6+^.

Spiked Value (mg/kg)	SERS			ICP-MS		
	Obtained Value (mg/kg)	Recovery Percentage (%)	RSD (%, n = 3)	Obtained Value (mg/kg)	Recovery Percentage (%)	RSD (%, n = 3)
2.5	2.621	104.84	2.06	2.528	101.12	1.07
5	4.686	93.72	1.39	4.8825	97.65	0.96
10	9.162	91.62	2.12	10.386	103.86	0.89
20	20.526	102.63	3.07	19.638	98.19	1.53

## Data Availability

The data presented in this study are available on request from the corresponding authors.
